# Coping, fostering resilience, and driving care innovation for autistic people and their families during the COVID-19 pandemic and beyond

**DOI:** 10.1186/s13229-020-00365-y

**Published:** 2020-07-22

**Authors:** Stephanie H. Ameis, Meng-Chuan Lai, Benoit H. Mulsant, Peter Szatmari

**Affiliations:** 1 Centre for Addiction and Mental Health, 80 Workman Way, Toronto, ON M6J 1H4 Canada; 2 Department of Psychiatry, The Hospital for Sick Children, Toronto, Canada; 3 Department of Psychiatry, Faculty of Medicine, University of Toronto, Toronto, Canada; 4 Autism Research Centre, Department of Psychiatry, University of Cambridge, Cambridge, UK; 5 Department of Psychiatry, National Taiwan University Hospital and College of Medicine, Taipei, Taiwan

**Keywords:** Autism, SARS-CoV-2 virus, COVID-19, Pandemic, Resilience, Telehealth, Health services, Equity

## Abstract

The new coronavirus disease (COVID-19) pandemic is changing how society operates. Environmental changes, disrupted routines, and reduced access to services and social networks will have a unique impact on autistic individuals and their families and will contribute to significant deterioration in some. Access to support is crucial to address vulnerability factors, guide adjustments in home environments, and apply mitigation strategies to improve coping. The current crisis highlights that our regular care systems are not sufficient to meet the needs of the autism communities. In many parts of the world, people have shifted to online school and increased use of remote delivery of healthcare and autism supports. Access to these services needs to be increased to mitigate the negative impact of COVID-19 and future epidemics/pandemics. The rapid expansion in the use of telehealth platforms can have a positive impact on both care and research. It can help to address key priorities for the autism communities including long waitlists for assessment and care, access to services in remote locations, and restricted hours of service. However, system-level changes are urgently needed to ensure equitable access and flexible care models, especially for families and individuals who are socioeconomically disadvantaged. COVID-19 mandates the use of technology to support a broader range of care options and better meet the diverse needs of autistic people and their families. It behooves us to use this crisis as an opportunity to foster resilience not only for a given individual or their family, but also the system: to drive enduring and autism-friendly changes in healthcare, social systems, and the broader socio-ecological contexts.

Autism spectrum disorder (ASD) is a relatively common neurodevelopmental condition [1] characterized by long-standing social communication challenges and restricted, repetitive, and inflexible patterns of behavior, interests, and activities (RRBI). Intensity of presentation, verbal and cognitive abilities, and adaptive functioning vary significantly across individuals on the autism spectrum and even within an individual over time. Autistic individuals (we acknowledge that some identify as, or prefer the terms “individuals with autism” or “individuals with ASD”) also experience high rates of co-occurring physical and mental health conditions (e.g., gastrointestinal and sleep problems, ADHD, anxiety, and depression) across the lifespan [2, 3], contributing to complexity and heterogeneity of presentation. Although all autistic individuals and their families require some forms of support [4], care needs vary considerably across individuals and across the lifespan. The new coronavirus disease (COVID-19) pandemic has changed how our local community and global society operate, impacting autistic individuals and their families, as well as autism clinical services and research progress [5]. An early analysis of globally federated electronic medical record data (through May 14, 2020) shows that individuals with intellectual and developmental disabilities (IDD, including autism) have higher prevalence of specific physical comorbidities associated with poorer COVID-19 outcomes. Furthermore, among younger people (≤ 17 years), those with IDD are more likely to be infected and experience higher fatality rate than those without IDD [6]. Within days of the World Health Organization declaring the pandemic, physical distancing measures were implemented, leading to the closure of schools and businesses, transition to work-from-home when possible, massive job loss, and economic uncertainty. With the aim of protecting physical health, such societal mitigation measures have inadvertently impacted the mental health and well-being of autistic individuals and their families, resulting in high stress, increased mental health challenges, and disrupted services and supports (e.g., see US-based survey report: https://sparkforautism.org/discover_article/covid-19-impact-asd/ & https://sparkforautism.org/discover_article/covid-19-and-its-impact-on-autistic-adults/). Early data from Italy have highlighted the negative impact to families with autistic children, including daily challenges managing structured activities and free times, and increased behavior regulation difficulties for the child (especially when behavioral challenges predate COVID-19) [7]. Turkish parents of autistic children also report higher levels of anxiety and lower feelings of dispositional hope and psychological well-being compared to parents of neurotypical children [8].

Autistic individuals and their families can be particularly impacted by the COVID-19 pandemic and the sequelae [9–11], especially those with intellectual disability or with high support needs [6, 12, 13]. For example, only 25% of autistic adults live in their own households [14], and many autistic adults with high support needs live in congregate care settings (e.g., long-term care facilities or group homes). The characteristics of these settings (i.e., shared bedrooms, bathrooms, and common space) and of their residents (e.g., with medical comorbidities, cognitive impairment, or dysregulated behavior) may impede traditional measures to prevent the spread of the virus (e.g., physical distancing and frequent hand-washing) [15, 16]. In addition, staff working across facilities can also accelerate the spread. Whether autistic individuals’ health and developmental vulnerability interacts with the pathophysiology of, and host responses to, the SARS-CoV-2 virus is not yet known, neither is its long-term developmental impact on the body and the brain [17]. The current pandemic is likely to have prolonged course and impact [18]. Similar epidemics and pandemics are also possible in the future. Therefore, it is essential to monitor the short-term and long-term challenges for autistic people and their families [10], reflect on the unique challenges that autistic individuals and their families are facing during the pandemic, and consider opportunities to support coping moving forward.

## The impact of environment changes and disrupted routines during the COVID-19 pandemic

The person-environment fit is crucial to the functioning and well-being of autistic individuals [4], as are parent-child interactions [19, 20]. The pandemic is changing several factors closely associated with the person-environment fit. These factors include balancing autistic individuals’ needs for routine and environmental predictability versus the unpredictability associated with the pandemic; family members’ understanding of autistic individuals’ social and sensory needs versus autistic individuals’ understanding of family situation and family members’ experiences; autistic individuals’ learning or working styles versus available opportunities at this time; and the living environment or activity schedules among family members. Strong needs for sameness and adherence to routines are core features of autism. They have been linked to the high rates of anxiety and depression experienced by autistic individuals [21]. Changes to everyday routines and restrictions to regular service disrupt a number of domains (e.g., physical health, mental health, and familial factors). For example, reduced access to specific foods will impact autistic individuals with selective eating [22], potentially leading to reduced food intake, poorer nutrition, or worsening elimination problems and constipation. Restricted access to regular programming, preferred activities and places contributes to lower physical activity, which is already a concern among autistic individuals, as are higher rates of obesity [23]. Home confinement also may lead to more time spent pursuing special interests. Although motivation for special interests is linked to well-being and satisfaction in autistic individuals, very high intensity engagement has detrimental effects on these domains [24]. During this pandemic, it is particularly challenging to regulate time spent on screens, which is already increased in autistic children [25]. All these factors may contribute to the exacerbation of problematic RRBIs [26], sleep problems [27], and aggression (ranging from verbal to physical) with high rates of direction towards caregivers [28]. We expect the pandemic to particularly affect autistic individuals with more behavioral difficulties, who already (i.e., prior to COVID-19 pandemic) experience more dysregulation of sleep, attention, anxiety, and depressive symptoms and more often take psychotropic medications [29]. Thus, to mitigate the effects of the pandemic on these at-risk autistic individuals will require complex physical and mental healthcare, including psychosocial and behavioral supports for both the individual [29] and their caregivers, who are also expected to experience elevated stress and anxiety [30].

Because of the risks associated with living in congregate settings discussed above, some autistic individuals have moved back to living with their family members. Others may no longer be able to visit their family due to distancing measures. All these environmental modifications may change sensory input (e.g., due to louder or different noise at home) and increase stress and anxiety among autistic individuals and their families. However, these challenges can be mitigated if the person-environment fit at home is comfortable. For instance, exposure to environments that cause social overload or sensory discomfort may be lowered with the judicious implementation of physical distancing measures necessitated by the pandemic. With gradual reopening of schools and businesses occurring after the first wave of the pandemic across many parts of the world, there is the potential that precautions will be tightened again to reduce virus spread and burden on medical systems with subsequent waves. The need to transition back and forth will be particularly difficult for autistic children and adults. This reality means that our care systems need to remain open, accessible, and flexible in response to the evolving crisis.

## The impact of school closure, shift to online schooling, and physical distancing during the COVID-19 pandemic

Schools often provide opportunities for interventions including specialized education, mental health care [31], and social interaction; they are critical for regulation of behaviors. On the other hand, school can also be a highly challenging setting for some autistic individuals due to academic difficulties, co-occurring ADHD or anxiety [2], and high risk of school and cyber-bullying from peers [32, 33]. All of these factors contribute to trauma, depression, anxiety, and suicidality [32, 34]. Thus, closure of schools (and work environments for adults) may result in reduced stress in some autistic individuals and increased stress in others [9]. When the person-environment fit is poor at home, school can be an outlet for young autistic individuals and their only option for social interaction. For autistic youth and adults, social interactions outside the school or work settings are already limited [35]. Therefore, physical distancing measures will further reduce access to this limited social support network (which is particularly crucial for adolescents and young adults [36]), potentially escalating mental health challenges (e.g., depression and suicidal ideation) [34] and have far-reaching consequences.

It is not yet known how the move toward online classrooms and distance learning during the pandemic will impact autistic individuals. For some, the freedom to be in their home environment with a self-paced schedule will have positive effects on learning outcomes and allow them to engage in RRBIs that are soothing and anxiety-relieving without negative social consequences. For others, a lack of one-on-one professional support in a structured, regulated environment, and away from distraction will be detrimental to learning. Making use of online platforms for education (or other services) will be the most difficult for those with high support needs, where communication and cognitive factors are critical barriers for engagement [37]. Provision of specialized education to support students with disabilities is legally mandated in many jurisdictions. However, access and quality are highly variable at the best of times (e.g., see [38] for a US example). This variability will increase with the added complexity of remote delivery. Online education also depends on digital access and caregiver availability in the home to support students, placing constraints on caregivers’ work [39]. Thus, the pandemic will amplify academic challenges, socioeconomic disadvantages, and parental burden for many autistic children and their families.

## The impact of COVID-19 pandemic on autism diagnostic services

The first wave of the COVID-19 pandemic and consequent mitigation measures (e.g., the closure of outpatient services) have resulted in challenges to autism diagnostic practices, which traditionally include in-person, interaction-based behavioral observation as a key component. In many jurisdictions, a formal autism diagnosis is required for a child to receive timely intervention and support. Thus, physical distancing measures may result in additional delays in formal diagnosis, compounding the current long waitlists for diagnostic assessments in many areas. These delays will deprive autistic children from timely support (e.g., early intervention, educational plan, and tailored medical care) that is needed to facilitate their development, health, and well-being. To mitigate this challenge, many diagnostic practices (particularly those for young children) have started to incorporate video-based naturalistic and caregiver-mediated behavioral observation (e.g., the Systematic Observation of Red Flags [40]), telehealth-based observation and interview [41], or are developing video-based observation and coding systems with remote administration of semi-structured activities by a parent or adult assessor based on the Autism Diagnostic Observation Schedule and the Brief Observation of Social Communication Change (e.g., the Brief Observation of Symptoms of Autism, BOSA, which is not yet validated as of July 9, 2020: https://www.semel.ucla.edu/autism/video/brief-observation-symptoms-autism-bosa-training). Although some virtual assessments for autism have shown preliminary validity [41, 42], they are designed primarily for young children. Comparable assessments have not been validated in older children, youth, or adults. The diagnostic challenges highlighted by the pandemic are forcing our field to re-evaluate the “best practices” for diagnostic assessments for autism when considering accessibility, timeliness, quality, and comprehensiveness. They also highlight the need to decouple eligibility for services from a “confirmed” or “formal” autism diagnosis and consider providing intervention based on individual needs, developmental level, function, and symptoms.

## Mitigation strategies during the acute phase of disruption associated with the COVID-19 pandemic

The pandemic creates ongoing and possibly long-lasting challenges. For autistic individuals and their families, short-term and medium-term mitigation strategies are as important as long-term prospects. We need to implement mitigation strategies quickly and learn from this experience to improve quick shifts to “new normal” care practices in the future. Maintenance of typical routines is impossible during the COVID-19 pandemic. However, the impact of changes in routines can be attenuated by jointly creating and implementing alternative routines, incorporating a regular bedtime and morning schedule, attention to sensory stimulation, adequate but limited exposure to media, regulated screen time, and attention to regular hygiene, food and water intake, daily exercise, and sleep. Maintaining social networks (even if the only viable option to do so is online) is critical for both autistic individuals and their families.

Ensuring access to adequate resources (e.g., education, healthcare, autism-specific support, financial support, parental support) should be a priority at this time, even if the majority of care resources must shift to virtual/remote delivery. Ongoing provision of care is critical for autistic people and their families to maintain stability and prevent deterioration of health and its associated burden on broader healthcare systems. Over the past two decades, psychotropic prescription rates have been increasing in children diagnosed with ASD [43, 44], with high rates of polypharmacy [44], and use of a variety of medication classes despite a lack of evidence [43, 44]. Tolerability of these psychotropic medications is a concern [45]. Although clinicians need to be mindful of the risk of over-prescribing medications that may contribute to poorer health in the long-term [46], clinicians will need to provide careful and timely intervention, targeting symptom reduction through pharmacological and non-pharmacological support to prevent crises and hospitalizations and facilitate environmental adjustments. As a result, it is crucial to maintain ongoing access to clinicians who provide care, support, and psychoeducation. They will need to address, in kind and compassionate ways, vulnerability factors, home environment, coping with current restrictions, implementation of mitigation strategies, and resources for dealing with stress. To date, our early experience has been mostly positive when providing mental health care remotely to verbal children, adolescents, and adults on the autism spectrum. Many autistic individuals are less stressed in their home environments without the need to travel to a clinic or hospital and manage sensory and social communication inputs in an unfamiliar and sometimes insensitive setting (e.g., in-person care interruptions due to hospital-wide overhead announcements). Some individuals seem to be able to communicate more effectively using telehealth platforms (via videoconference or telephone delivery), either verbally or with chat boxes. Clinicians also benefit from being able to see the home setting and to witness the interaction at home between parent and child or among household members including pets. Nevertheless, direct telehealth care may not work well for minimally verbal autistic individuals or those having difficulties adapting to virtual platforms. In these scenarios, support will need to be delivered in alternative ways (e.g., telehealth care delivered to and mediated by parents or family members).

A number of helpful COVID-19 online resources from local and global professional organizations have been collated to support care at this time (see Table 1). This growing list highlights the value of the internet in rapidly distributing practical information and offering timely mitigation strategies for autistic individuals and their families.

**Table 1:** Examples of COVID-19-related online resources

**General resources**	
World Health Organization	https://www.who.int/campaigns/connecting-the-world-to-combat-coronavirus/healthyathome
World Psychiatric Association	https://www.wpanet.org/covid-19-resources
COVIDwithKIDS	https://www.covidwithkids.org
**Autism-specific resources**	
US Department of Health & Human Services, Interagency Autism Coordinating Committee (IACC)	https://iacc.hhs.gov/resources/coronavirus/
Autism Speaks	https://www.autismspeaks.org/covid-19-information-and-resources
Autism Science Foundation	https://autismsciencefoundation.org/covid-19-resources/
Autism Society of America	https://www.autism-society.org/covid-19/
UK National Autistic Society	https://www.autism.org.uk/services/helplines/coronavirus.aspx
Canadian Autism Spectrum Disorders Alliance	https://www.casda.ca/resources/covid-19-navigation-guide/
Autism Spectrum Australia	https://www.autismspectrum.org.au/how-can-we-help/covid-19-information
UCLA Center for Autism Research & Treatment	https://www.semel.ucla.edu/autism/covid-19-resources
Health Care Access Research and Developmental Disabilities Program (H-CARDD)	https://www.hcarddcovid.com/info

## Opportunities to foster resilience and improve care for autistic individuals and their families during and after the COVID-19 pandemic

Despite the challenges of the current pandemic, our collective experiences with coping may cultivate resilience and reveal opportunities for creating more flexible and better care in the future. Resilience is a process of interactive adaptation that facilitates coping in the face of adversity linked with a person’s neurological and psychological makeup [47], and, equally (if not more) importantly, the socio-ecological contexts [48]. Past research in families of autistic individuals has shown that resilience is associated with social support, coping style (e.g., problem-focused coping), cognitive appraisal, optimism, locus of control, self-efficacy, acceptance, sense of coherence, and positive family function [49, 50]. Autistic individuals demonstrate their resilience everyday through coping despite heightened stress and anxiety due to uncertainty and limited opportunities for in-person social interaction [9]. The knowledge about factors fostering resilience is critical to improve care and support for autistic individuals and their families [51] both during and after the pandemic. These factors should be considered in the context of the unique sets of socio-ecological and psychological stressors experienced by autistic people and their families during the pandemic, e.g., socio-interpersonal challenges during lockdown, accessibility to healthcare, and vulnerability to job loss and employment crisis [10].

Nonetheless, the disruption of an already fragile system of care during the pandemic could substantially worsen the mental health of autistic individuals and reduce access to care, and some of this worsening may not be reversible. Current system limitations compounded by the pandemic are particularly concerning in young children. The greatest gains of early interventions are made in autistic children who have begun to make progress by 3 years of age [52]. For those most disadvantaged by socio-cultural factors, access to basic services may not be available within an acceptable timeframe even at the best of times [53]. As autistic individuals grow up, their care needs change. Autistic youth and young adults have more emergency department visits, more primary and psychiatric care visits, and higher healthcare expenditures than other youth with special healthcare needs [54, 55]. Rates of complex mental health conditions (e.g., depression, schizophrenia spectrum disorders, and bipolar disorders) are more prevalent in autistic individuals than in the general population, and they increase with age [2]. Optimal care needs to be multidisciplinary, bringing autistic individuals, their families, and care providers together, to facilitate a shared decision-making process and personalized care delivered at the right time [4]. The COVID-19 pandemic has highlighted and magnified many existing and new problems in our service and care systems for autistic individuals and their families. Coping with the pandemic offers an opportunity for change and fostering the resilience of autistic individuals, families, service providers, and the overall care systems.

## Opportunities to use virtual platforms to deliver care for autistic individuals and their families

Priorities enumerated by the autism communities include improved service delivery, development of person-centred interventions, improved education of family members to support and understand their autistic relatives, improved support for co-occurring mental health conditions and well-being, and research supporting adult transitions and lifespan issues [56] (also see: http://www.jla.nihr.ac.uk/priority-setting-partnerships/autism/top-10-priorities/). Insufficient funding has impeded care delivery for autistic individuals within current healthcare service structures [57]. The rapid increase in the use of telehealth and virtual platforms for remote assessment and care delivery could have a positive impact on these key priorities for the autism communities. A growing evidence base supports the feasibility and effectiveness of tele-mental health care [31, 58]. Virtual care can also address the issues of long waitlists, limited access in remote locations, restricted hours of service, and “no-show” rates [59]. Preliminary data have shown these benefits in autism diagnostic services for young children [60]. Virtual care could also reduce barriers to accessing care due to stigma [58].

### Technology to implement evidence-based interventions

In young children with ASD, a shift toward using technological platforms could have a lasting impact on care delivery. Parent involvement in early intervention for young children can have positive long-term effects [4]; it predicts IQ into adulthood, achievement, and adaptive functioning [61]. Even low-cost, low-intensity parent-mediated interventions involving coaching parents around interacting with their young children with ASD can have immediate effects on social behavior and communication [62]. Increasing evidence suggests that caregivers could be trained remotely to deliver parent-mediated interventions, and remote training can impact parent knowledge, intervention fidelity, and social behavior or communication skills of children [63, 64]. A rapid shift toward remote options to implement caregiver-mediated interventions could have an important impact. For example, in response to the COVID-19 pandemic, the Early Start Denver Model team has created online education modules to enhance caregiver-mediated early intervention (https://helpisinyourhands.org/course). Online resources for telehealth-based assessment and service delivery are also accumulating (e.g., https://autismnavigator.com, https://triad.vkclearning.org). Additional telehealth-based support for early intervention (via caregivers) and targeted intervention for autistic children, youth, and adults should be developed and tested via remote clinical trials [65]. This would substantially enhance the accessibility to service and support even beyond the COVID-19 pandemic.

### Technology to facilitate coordinated multidisciplinary care

Virtual platforms that do not require a team to be in the same place to deliver coordinated care can improve complex care delivery for autistic individuals and their families. As described by the American Academy of Pediatrics, the medical home concept can be implemented for a variety of general medical and psychiatric conditions; it includes a comprehensive centralized health record, culturally competent care, and interaction across schools and service providers to support families [66]. The model described for autistic individuals emphasizes care coordination and communication between families, clinical teams, community agencies, and education or employment settings [67].

### Technology to support a broader range of care options and early stages of stepped care

The use of online platforms can change service delivery away from one-size-fits-all approaches [68]. It can ensure that more autistic individuals have access to care. It can support personalized and stepped care models (i.e., a system of delivering and monitoring support so that the most effective and least resource-intensive care is delivered first, with “stepping-up” to intensive services as required) where a variety of intervention approaches are available. Online platforms (e.g., https://autismnavigator.com) are particularly useful for large-scale access to earlier stages of care including psychoeducation, self-directed (or caregiver-directed), or minimally supported interventions (e.g., periodic check-ins to support self-directed interventions). These platforms may also be useful to provide care for mild to moderate anxiety or depression [68].

### Ensuring equity and accessibility to improved virtual model of care should be a priority for health service systems

The success of rapid expansion of telehealth for autistic individuals depends on a number of factors. They include flexible funding that provides equivalent remuneration for in-person and remote care [58]. Socioeconomic factors contributing to differential access also need to be considered. Currently, richer and better educated people are much more likely to access and benefit from digital care [69]. Some caregivers cannot access remote care while juggling the added workload imposed upon them during the pandemic. While we develop virtual care to improve access, we must be mindful that it could worsen care inequity and variability [31, 70]. We also need to develop alternative models to support autistic individuals and caregivers unable to access virtual care (e.g., distanced in-person visits). The COVID-19 pandemic emphasizes issues of inequity. Health professionals need to advocate for equalizing access and adopting a “digital health equity framework” [70].

## Every cloud has a silver lining

US-based surveys of about 8,000 *SPARK* parents or guardians and 600 autistic adults in March–April 2020 reveal some positive experiences coping with the pandemic, despite high stress and disruption: positive use of time, extra family time and time to pursue hobbies, limiting social media and news, exploration of calming activities, slower pace for learning, and adaptation to virtual social connection, learning, and healthcare (see: https://sparkforautism.org/discover_article/covid-19-impact-asd/ & https://sparkforautism.org/discover_article/covid-19-and-its-impact-on-autistic-adults/); however, it is likely that the respondents of these surveys have better resources, and they may not be representative of the autism population at large. An *Autistica* live article indicates that global changes required by the pandemic can lead to better understanding and accommodation for autistic people in the medium and long-term, including more flexible and empathetic workplaces; improved access to resources, events, and therapeutic support that can be offered remotely; and embracing innovative and more diverse teaching and learning opportunities (https://www.autistica.org.uk/news/world-after-coronavirus). More lessons will be learned and innovations developed in the coming months for autistic individuals and their families [5]. In the long-term, this pandemic could foster resilience not only for the autism communities and the stakeholders, but also for the professionals who provide care. The current crisis could serve as a *turning point* to change the health and social systems and the broader socio-ecological contexts to be more flexible, empathetic, and autism-friendly. Once the pandemic is resolved, useful new models of care can be maintained and generalized instead of reverting back to the business-as-usual pre-pandemic state. The pandemic is an opportunity for *stress inoculation* [51]: if we can make the COVID-19 experiences manageable, we are better prepared for other crises in the future.

## Data Availability

Not applicable

## Abbreviations

ADHD: Attention-deficit/hyperactivity disorder
ASD: Autism spectrum disorder
COVID-19: Coronavirus disease 2019
RRBI: Restricted, repetitive, and inflexible patterns of behavior, interests, and activities
